# Process Characterization by Definitive Screening Design Approach on DNA Vaccine Production

**DOI:** 10.3389/fbioe.2020.574809

**Published:** 2020-10-15

**Authors:** Lalintip Hocharoen, Sarawuth Noppiboon, Panit Kitsubun

**Affiliations:** ^1^Bioprocess Research and Innovation Centre (BRIC), National Biopharmaceutical Facility (NBF), King Mongkut’s University of Technology Thonburi (KMUTT), Bangkok, Thailand; ^2^Biochemical Engineering and System Biology Research Group (IBEG), National Center for Genetic Engineering and Biotechnology (BIOTEC), National Science and Technology Development Agency (NSTDA), Bangkok, Thailand

**Keywords:** process characterization, DNA vaccine, definitive screening design, critical process parameter, critical quality attributes

## Abstract

Plasmid DNA is a vital biological tool for molecular cloning and transgene expression of recombinant proteins; however, decades ago, it has become an exceptionally appealing as a potential biopharmaceutical product as genetic immunization for animal and human use. The demand for large-quantity production of DNA vaccines also increases. Thus, we, herein, presented a systematic approach for process characterization of fed-batch *Escherichia coli* DH5α fermentation producing a porcine DNA vaccine. Design of Experiments (DoE) was employed to determine process parameters that have impacts on a critical quality attribute of the product, which is the active form of plasmid DNA referred as supercoiled plasmid DNA content, as well as the performance attributes, which are volumetric yield and specific yield from fermentation. The parameters of interest were temperature, pH, dissolved oxygen, cultivation time, and feed rate. Using the definitive-screening design, there were 16 runs, including 3 additional center points to create the predictive model, which then was used to simulate the operational ranges for capability analysis.

## Introduction

Gene immunization including DNA vaccines has become an attractive approach for vaccination because of its well-documented safety unlike live attenuated viral vaccine, the absence of specific immune responses to the plasmid, and its absence of genetic integration (Robinson, 2000; Wahren and Liu, 2014). DNA vaccination is genetically engineered DNA containing a transgene that expresses a specific antigen into cells or tissues (Ingolotti et al., 2010). To date, there are six DNA vaccines approved for veterinary applications, which include preventive vaccines for West Nile virus infection in horses (Davidson et al., 2005), hematopoietic necrosis virus infection in salmon (Garver et al., 2005), therapeutic cancer vaccine for dogs (Bergman et al., 2006), a growth hormone gene therapy to increase litter survival in breeding pig sows (Person et al., 2008), pancreas disease infection in Atlantic salmon (EMA, 2017), and H5N1 in chicken with conditional license (Agrilabs, 2017). There are also a number of DNA vaccines undertaking clinical studies for human uses including GX188E and VGX-3100 for human papillomavirus (Cheng et al., 2018), a prime/boost of DNA.Mel3 with MVA.Mel3 for advanced metastatic melanoma cancer treatment (Dangoor et al., 2010) and INO-4800 for COVID-19 (Smith et al., 2020).

The production process for DNA vaccine consists of several steps with aims of achieving high quantity and quality to meet product specifications. The US Food and Drug Administration recommends that at least 80% supercoiled content shall be obtained as this has superior biological activity as compared to other plasmid forms (Urthaler et al., 2005; U.S. FDA, 2007). Therefore, it is indispensable to demonstrate process robustness with identified critical process parameters (PPs) and critical quality attributes (CQAs) during the process development phase. This process characterization applies Quality by Design principle to help establish a rational and cost-effective approach on process design and optimization (ICH, 2009; McCurdy, 2011). By performing Design of Experiments (DoE), it is allowed to use a minimum number of experimental runs where all experimental parameters studied are varied simultaneously to obtain sufficient information (Mandenius and Brundin, 2008; Montgomery, 2017). Conventional DoE scheme is first via screening designs to determine significant main effects followed by response-surface models to justify the design space. This requires many experimental runs to gain sufficient data for further analysis, resulting in taking more times and resources (Abu-Absi et al., 2010; Erler et al., 2013). An alternative one-step design method named definitive-screening design (DSD) was introduced by Jones and Nachtsheim. This design method contains several desirable properties (Jones and Nachtsheim, 2011; Xiao et al., 2012; Erler et al., 2013; Nguyen and Stylianou, 2013; Tai et al., 2015);

1. offering the identification for main effects, quadratic effects, and two-factor interaction based on the sparsity-of-effects system,
2. providing an orthogonal model that the main effects are uncorrelated with two-factor interaction and quadratic effects, and two-factor interaction is not fully aliased with each other,
3. requiring a minimum number of runs as few as 2m + 1 or 2m + 3, for *m* variable factors when *m* is even and odd number, respectively.

DSD has become widely used in diverse applications including paint manufacturing, green energy, and biotechnology. In this study, we employed DSD method to characterize the process of porcine DNA vaccine production. The method involved five PPs at the *Escherichia coli* DH5α fermentation step to investigate the impact on CQAs, which is supercoiled content, and performance attributes (PAs), which are volumetric yield and specific yield. Finally, a simulation run accounting for variability expected in larger-scale production was executed to provide a prediction model. The relationships presented here were expected to demonstrate the robustness of the fed-batch fermentation process for subsequent process validation and future commercial manufacturing. The utilization of knowledge gained can then be used to improve the process performance during the process development.

## Materials and Methods

### Plasmid and Host Strain

*E. coli* DH5α [F- Φ 80dlacZΔM15 Δ(lacZYA -argF) U169 recA1 endA1 hsdR17 (rk-, mk+) phoA supE44 λ-thi-1 gyrA96 relA1] containing pTH.PRRSV_GP5 plasmid was kindly provided by Dr. Yaowaluck Maprang Roshorm, School of Bioresources and Technology, King Mongkut’s University of Technology Thonburi. The plasmid is encoded for porcine reproductive and respiratory syndrome (PRRS) virus antigen, which will be used as a porcine DNA vaccine. The construct of this plasmid is reported in a conference paper by Pannacha (2014).

### Inoculum and Fermentation

Inoculum was cultured in 20 mL Luria broth overnight at 37°C with agitation at 200 rpm (New Brunswick Innova 43R, United States) and transferred to a main cultivation in 2 L fermentor (Sartorius BIOSTAT B Plus, Germany) with semidefined media that comprised 3 g/L KH_2_PO_4_, 6 g/L Na_2_HPO_4_, 2 g/L NH_4_Cl, 1 g/L MgSO_4_, 20 g/L yeast extract, and 5 g/L glycerol. The starting volume was 0.7 L, and an initial OD_600_ was set around 0.02. Batch fermentation was performed with set points at 37°C, pH 7, 30%DO, and 1 vvm air flow rate. When the glycerol in the culture was totally consumed, the fed-batch was started with 200 g/L glycerol. The feed rate was at 3 mL/h and linearly increased varying from 0 to 0.6 mL/h/h, depending on the designed experimental runs. Set points for PPs including temperatures, pH, %DO, cultivation time, and feed rate were varied with regard to DSD as described in section “Design of Experiment”.

### Design of Experiment

With prior knowledge and risk assessment tools, CQAs and PAs for DNA vaccine production were identified and led to a generation of a list of PPs with their associated ranges shown in Table 1. Five PPs for fed-batch fermentation including temperature, pH, dissolved oxygen, feed rate, and cultivation time were investigated. Sixteen experimental runs designed by DSD using JMP Pro software (SAS Institute Inc., Cary, United States) are listed in Table 2. Three replicate runs at the center points were also included in order to better estimate the error of experiments.

**TABLE 1:** List of process parameters and their ranges.

**Symbol code**	**Process parameter**	**Experimental values**		
		**Low level**	**Middle level**	**High level**
A	pH	6.8	7.0	7.2
B (°C)	Temperature	35	37	39
C (%)	Dissolved oxygen	20	30	40
D (mL/h/h)	Feed rate	0	0.3	0.6
E (h)	Cultivation time	17	18	19

**TABLE 2:** Definitive-screening design (DSD) and experimental data responses.

**Run**	**Variable level**					**%SC pDNA**	**Volumetric yield (mg pDNA/L)**	**Specific yield (mg pDNA/L/OD600)**
	**A**	**B**	**C**	**D**	**E**			
1	7.2	39	40	0.0	18	73.27	101.72	3.51
2	6.8	35	20	0.6	18	77.40	67.32	2.28
3	6.8	39	30	0.6	17	78.10	117.68	3.80
4	7.0	37	30	0.3	18	79.45	118.76	5.26
5	7.2	39	20	0.3	17	76.15	96.96	3.52
6	6.8	37	40	0.0	17	76.88	85.16	3.15
7	7.0	37	30	0.3	18	76.90	103.40	5.06
8	6.8	35	40	0.3	19	70.18	64.56	2.36
9	6.8	39	20	0.0	19	73.85	92.72	3.06
10	7.0	39	40	0.6	19	76.73	121.60	4.07
11	7.2	37	20	0.6	19	80.35	73.12	3.29
12	7.0	37	30	0.3	18	78.13	86.20	3.37
13	7.2	35	30	0.0	19	75.26	90.00	2.84
14	7.2	35	40	0.6	17	77.02	91.72	3.02
15	7.0	37	30	0.3	18	76.50	117.20	4.52
16	7.0	39	40	0.6	19	76.53	98.08	4.44

### Predictive Model Building and Process Robustness Study

Predictive model was fitted with all possible models using JMP Pro software, and the model selection determining active effects were the corrected Akaike information criterion (AICc) and the Bayesian information criterion (BIC) (Burnham and Anderson, 2004). The relationship between the responses (*y*) and variable factors (*x*) can be described by using the following quadratic predictive model:

$$y_{i}=\beta_{0}+\sum_{i=1}^{m}\beta_{j}\,x_{i,j}+\sum_{j=1}^{m-1}\sum_{k=j+1}^{m}\beta_{j\,k}\,x_{i,j}\,x_{i,k}+$$

∑i=1mβj⁢jxi,j2+,ii=1,…, 2m+3

where β_*0*_ is constant; β_*j*_, β_*jk*_, and β_*jj*_ are regression coefficients for linear, interaction, and quadratic terms, respectively; and _*i*_ is error.

**FIGURE 1:**
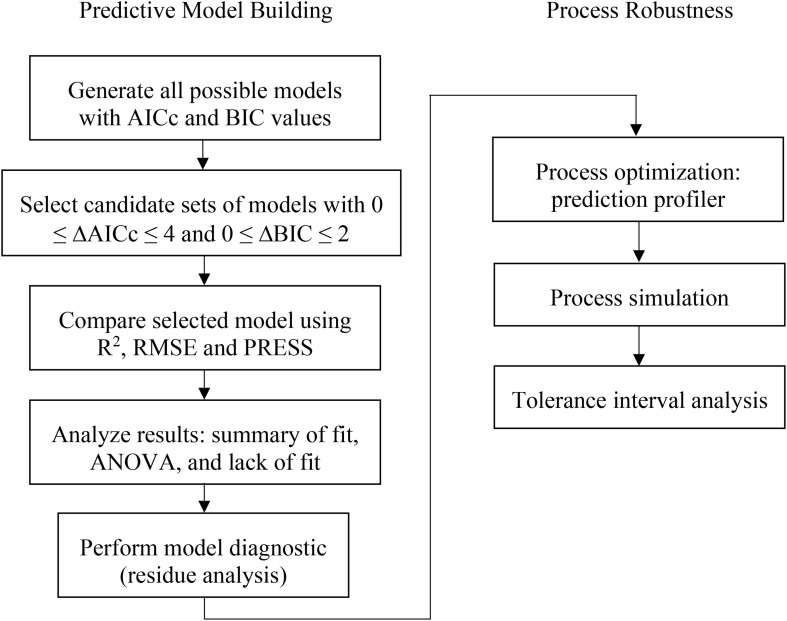
Predictive model building and process robustness diagram.

The model selection was evaluated using a combined AICc and BIC approach where models containing ΔAICc less than or equal to 4 and ΔBIC less than or equal 2 were selected. Then other statistical values, such as coefficient of determination (*R*^2^), predicted residual error sum of squares (PRESS), and root mean squared error (RMSE) were used as criteria to choose the best prediction model for each attributes. The prediction profiler function in JMP Pro software was then used for process optimization and simulation studies. The optimization was expected to provide the understanding of what factors highly influence the fermentation process of this vaccine production. For simulation study, due to a variety of distribution type, Monte Carlo simulations approach was conducted in 100,000 simulation runs. The results of this simulation provide the tolerance interval of the PAs, which can then be set as the action and alert limit, as well as provide the acceptable range on the CQA to establish the specification of the product. The algorithm based on the construction of all possible models with DSD is displayed in Figure 1.

### Sample and Sampling Preparation

Cell sample from each experimental run was taken at the end of batch for OD_600_ measurement, and DNA quantification and qualification. OD_600_ was measured by spectrophotometer (Eppendorf BioSpectrometer Kinetic, United States). The plasmid was extracted from 250 μL cell sample and followed by DNA extraction regarding the manufacturing’s protocol (QIAprep Spin Miniprep Kit, United States). DNA was then eluted with 100 μL elution buffer (10 mM Tris, pH 8.5). Plasmid DNA quantification was determined using A_260_ method (McGown, 2000; Stephenson, 2003). The volumetric yield (mg pDNA/L) calculation was conducted in which the dilution factor of amount sample taken and DNA elution were included. The specific yield of DNA (mg pDNA/L/OD600) was done by dividing the volumetric yield with the amount of cell, OD_600_. The supercoiled DNA content was determined on an agarose gel (0.7%) stained with ethidium bromide (0.5 μg/mL). As different DNA isoforms have distinct run patterns on agarose gels (Aaij and Borst, 1972), the supercoiled DNA band can be identified, and the ratio of supercoiled DNA was then calculated based on the band’s intensity using ImageJ software (Schneider et al., 2012).

## Results and Discussion

Prior knowledge on scientific understanding of DNA vaccine and risk assessment were used to select attributes. According to their impacts on clinical safety or efficacy and on manufacturing process, the form of plasmid DNA, % supercoiled DNA, referred to as %SC, directly affects the efficacy of DNA vaccines and hence is listed as one of the specifications given by regulatory (U.S. FDA, 2007) and consequently considered as CQA in this study. In order to evaluate how well the process performs, PAs has been introduced. They also relate to the process acceptable range that is used to ensure effectiveness of process performance and achievement of desired product specifications. For the purpose of our experiment, the volumetric yield, derived from DNA yield extracted at the end of each experimental batch and its corresponding OD600 value, and the specific yield of plasmid DNA were selected as our PAs.

DoE for *E. coli* DH5α fed-batch fermentation producing pTH.PRRSV_GP5 of five selected PPs was created to build the predictive models for DNA process production. Five PPs including temperature, pH, dissolved oxygen (%DO), cultivation time, and feed rate were evaluated. Using conventional approach, the experimental runs would require 16 runs for fractional factorial design and 46 runs for response surface design, whereas with DSD, the number of experimental runs was then reduced to 13 runs. This is the main advantage of DSD, an orthogonal model and free of aliasing with quadratic effects and two-way interactions. Therefore, DSD has been recommended for use in the earliest stage of the experimental study, where the numbers of potentially important variables are large, typically more than 4, to identify what highly influential factors are.

As mentioned above, this work aims to characterize the process of porcine DNA vaccine production particularly in the fermentation step. Thus, we chose DSD as a tool to design the experiment to better understand the potential important factors that may affect our interest attributes, PAs and CQA. With DSD, 13 runs were designed, and additional three replicate runs at the center points were taken into account for a better estimation on the error of experiments. The experimental results are shown in Table 2 where substantial variation ranges from 70.18 to 80.35 for %SC, 64.56 to 121.60 mg/L for volumetric yield, and 2.28 to 5.26 mg/L/OD600 for specific yield.

**FIGURE 2:**
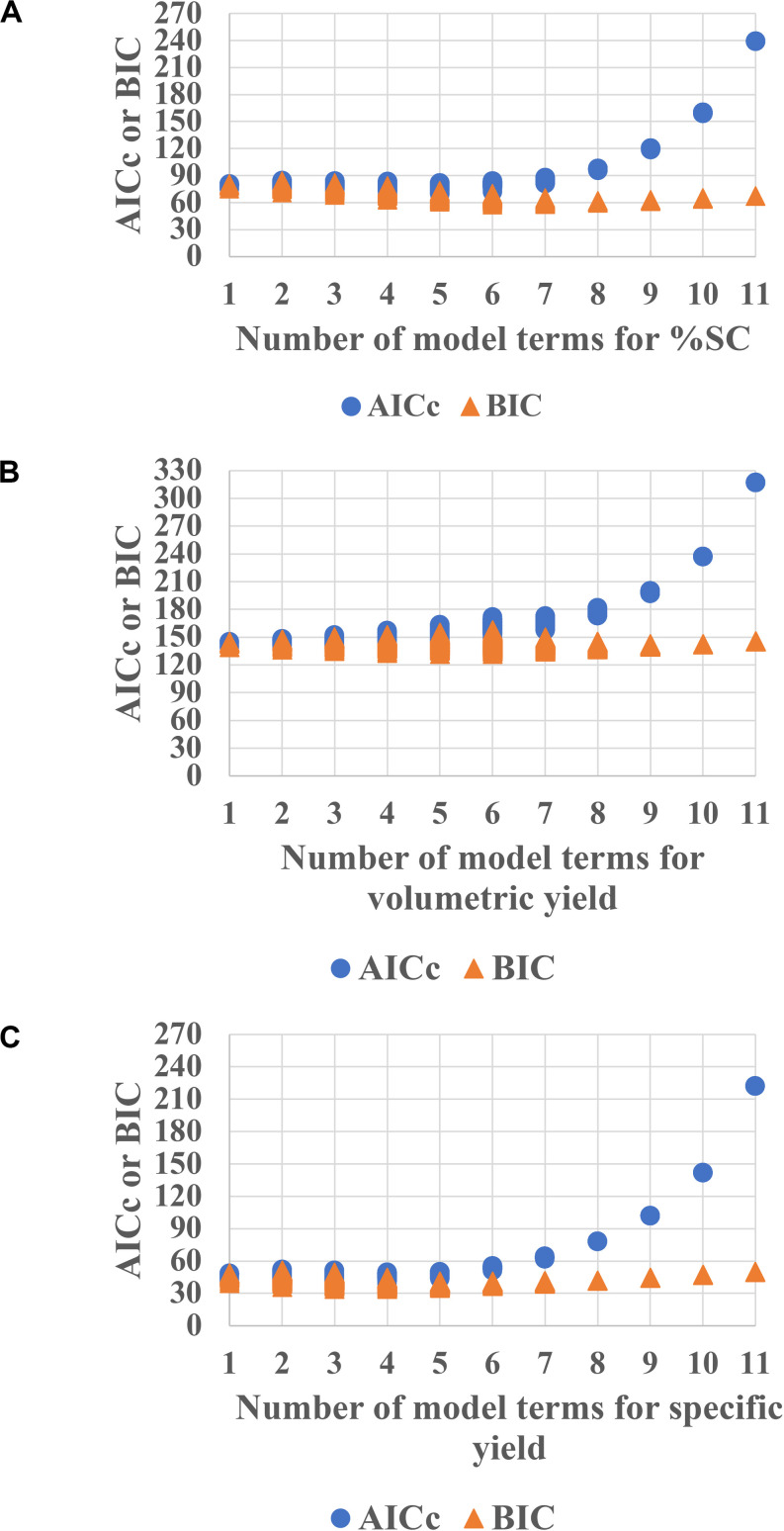
AICc and BIC plots for each attribute **(A)** %SC, **(B)** volumetric yield, and **(C)** specific yield for *E. coli* pTH.PRRSV_GP5. The lower values of AICc and BIC indicate better model prediction. Hence, models with the number of term of 4–6 are expected to provide sufficient prediction capability for %SC, whereas 4–6 terms and 3–5 terms are for volumetric yield prediction and specific yield models, respectively.

The data were then fitted using JMP Pro software. Generally, the model estimates only the main effects, two-factor interaction and quadratic. Therefore, with the PPs of 5, there is a total of 21 terms that is estimated including 5 main effects, 5 quadratic effects, 10 two-factor interaction, and 1 intercept. However, for DSD, the maximum number of terms that can be estimated is 11 because of the principle of effect sparsity. Then, subsets of all possible models with information criterion theory were used to investigate which model consists of the active effects (Ward, 2008; Mangan et al., 2017). The AICc and the BIC are one of the most used approaches for model prediction. These AICc and BIC calculations measure the model performance in which the smaller values indicate better model prediction (Burnham and Anderson, 2004). Herein, the candidate sets of models were generated with each attribute displayed in Figure 2. The models with the number of term of 4–6, showing the lowest AICc or BIC, are expected to provide sufficient prediction capability for %SC (Figure 2A), whereas 4–6 terms (Figure 2B) are for volumetric yield prediction, and 3–5 terms (Figure 2C) are for specific yield models.

**FIGURE 3:**
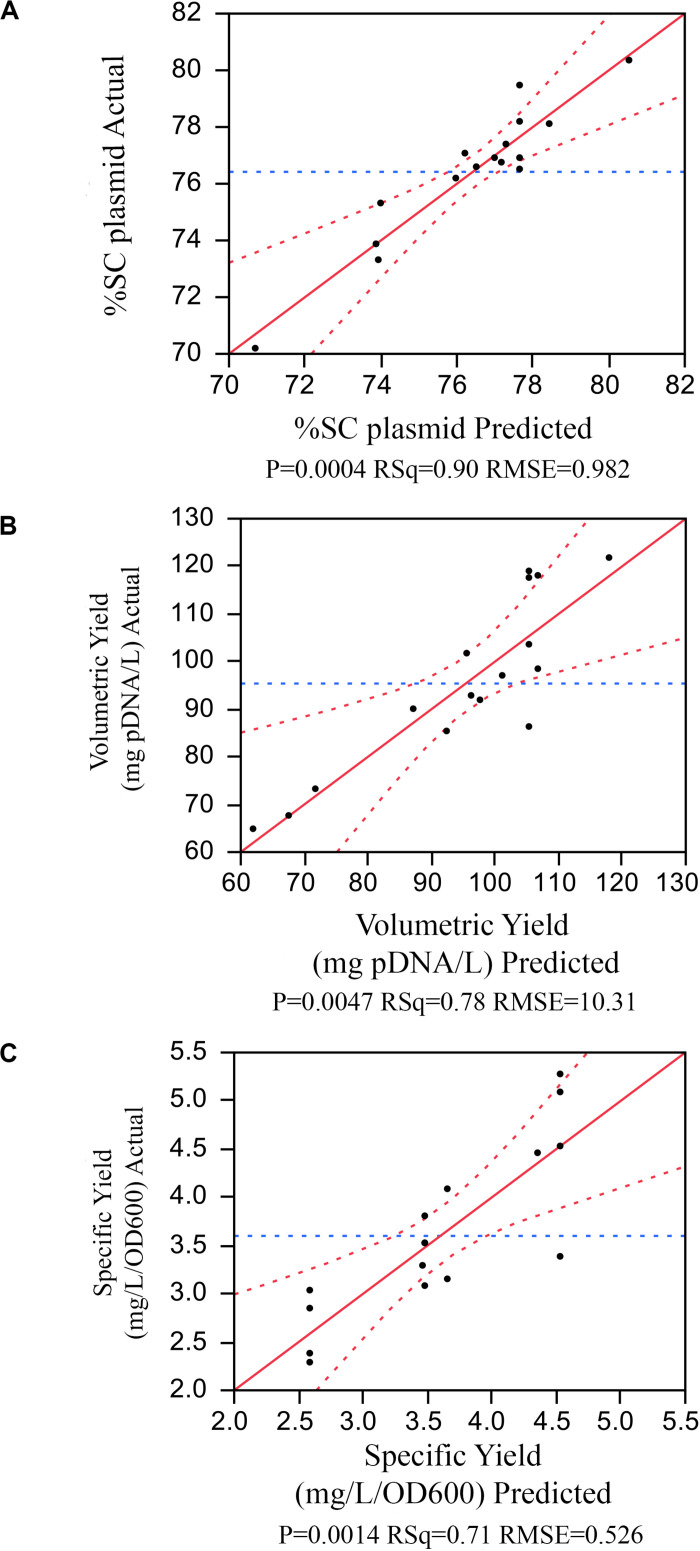
The prediction plot by the actual plot for **(A)** %SC, **(B)** volumetric yield, **(C)** specific yield.

The model selection was further considered using criteria of combined AICc and BIC, where ΔAICc was less than or equal to 4, and ΔBIC was less than or equal 2, and then using statistical values including *R*^2^, PRESS, and RMSE. As a result, in Figure 2A, the model with the number of terms = 6 was selected for %SC attribute. This 6-term prediction model included the main effects of %DO, feed rate, and cultivation time; the quadratic effects of pH; and two-factor interaction of pH and cultivation time, as well as temperature and cultivation time. The selected models provided a good description of the process as shown in the prediction plot by the actual plot in Figure 3A with *R*^2^ of 0.90, and the predictive models were highly significant with no evidence of lack of fit listed in Table 3.

**TABLE 3:** Regression analysis of predicted models for %SC.

**Analysis of variance**					
**Source**	**DF**	**Sum of squares**	**Mean square**	***F* ratio**	**Prob > *F***
Model	5	3,728.1972	745.639	7.0046	0.0047*
Error	10	1,064.4995	106.450		
C. Total	15	4,792.6967			
**Lack of fit**					
Lack of fit	7	378.0503	54.007	0.2360	0.9472
Pure error	3	686.4492	228.816		
Total error	10	1,064.4995			

**Parameter estimates**						
**Term**	**Scaled estimate**	**Std error**	***t* Ratio**	**Prob > |*t*|**	

Intercept	77.690803	0.42134	184.39	<0.0001*	
%DO (20, 40)	−1.06	0.310669	−3.41	0.0077*	
Feed rate (0, 0.6)	0.617	0.310669	1.99	0.0783	
Cultivation time (17, 19)	−1.595	0.310669	−5.13	0.0006*	
pH × pH	−2.035285	0.547765	−3.72	0.0048*	
pH × cultivation time	1.4356477	0.360583	3.98	0.0032*	
Temperature × cultivation time	0.8575907	0.387328	2.21	0.0541	

*Identified variable with a significant effect on response (*p*-value < 0.05).

Similar approach was applied to other attributes. The volumetric yield model with five terms contained the main effect of pH, temperature, and feed rate; the quadratic effects of temperature; and the two-factor interaction of pH and temperature. The specific yield model had three active terms consisting of the main effect and quadratic effect of temperature, and the quadratic effect of pH. All predictive model illustrated in the prediction plot by the actual plot (Figures 3B,C) and regression analysis (Tables 3–5) showed low F ratio of lack of fit, indicating that these models can be used for predicting the results. Hence, three quadratic models on %SC, volumetric yield, and specific yield, respectively, are illustrated below:

**TABLE 4:** Regression analysis of predicted models for volumetric yield.

**Analysis of variance**					
**Source**	**DF**	**Sum of squares**	**Mean square**	***F* ratio**	**Prob > *F***
Model	5	3728.1972	745.639	7.0046	0.0047*
Error	10	1,064.4995	106.450		
C. total	15	4,792.6967			
**Lack of fit**					
Lack of fit	7	378.0503	54.007	0.2360	0.9472
Pure error	3	686.4492	228.816		
Total error	10	1,064.4995			

**Parameter estimates**						
**Term**	**Scaled estimate**	**Std error**	***t* Ratio**	**Prob > |*t*|**	

Intercept	105.38667	4.212085	25.02	<0.0001*	
pH (6.8, 7.2)	7.456	3.262667	2.29	0.0454*	
Temperature (35, 39)	12.044	3.262667	3.69	0.0042*	
Feed rate (0, 0.6)	5.368	3.262667	1.65	0.1309	
pH × temperature	−7.695	3.647772	−2.11	0.0611	
Temperature × temperature	−15.99867	5.327913	−3.00	0.0133*	

*Identified variable with a significant effect on response (*p*-value < 0.05).

**TABLE 5:** Regression analysis of predicted models for specific yield.

**Analysis of variance**					
**Source**	**DF**	**Sum of squares**	**Mean square**	***F* ratio**	**Prob > *F***
Model	3	8.235242	2.74508	9.9108	0.0014*
Error	12	3.323750	0.27698		
C. total	15	11.558992			
**Lack of fit**					
Lack of fit	5	0.8524095	0.170482	0.4829	0.7799
Pure error	7	2.4713402	0.353049		
Total error	12	3.3237496			

**Parameter estimates**						
**Term**	**Scaled estimate**	**Std error**	***t* Ratio**	**Prob > |*t*|**	

Intercept	4.5313165	0.238023	19.04	<0.0001*	
Temperature (35, 39)	0.4535834	0.166427	2.73	0.0184*	
pH × pH	−0.874411	0.307286	−2.85	0.0147*	
Temperature × temperature	−0.616318	0.307286	−2.01	0.0680	

*Identified variable with a significant effect on response (*p*-value < 0.05).

%SC=77.69-1.06(%DO)+0.62(feedrate)-1.60⁢(cultivation⁢time)+0.86⁢(temperature)

(cultivation⁢time)-1.44⁢(pH)

$$\left(\mathrm{cultivation}\mathrm{time}\right)-2.04\left(\mathrm{pH}\right){}^{2}$$

Volumetric⁢yield=105.39+12.04⁢(temperature)+7.46⁢(pH)+5.37⁢(feed⁢rate)-7.70⁢(pH)

$$\left(\mathrm{temperature}\right)-16.00\left(\mathrm{temperature}\right){}^{2}$$

Specific⁢yield=4.53+0.45⁢(temperature)-0.62(temperature)-20.87(pH)2

With these optimized PPs; temperature of 38°C, pH 7, 20 %DO, feed rate of 0.6 mL/h/h and 17-h cultivation time, %SC plasmid DNA of 80.53 and volumetric yield of 112.78 mg pDNA/L and specific yield of 4.6 mg pDNA/L/OD600 were obtained.

**FIGURE 4:**
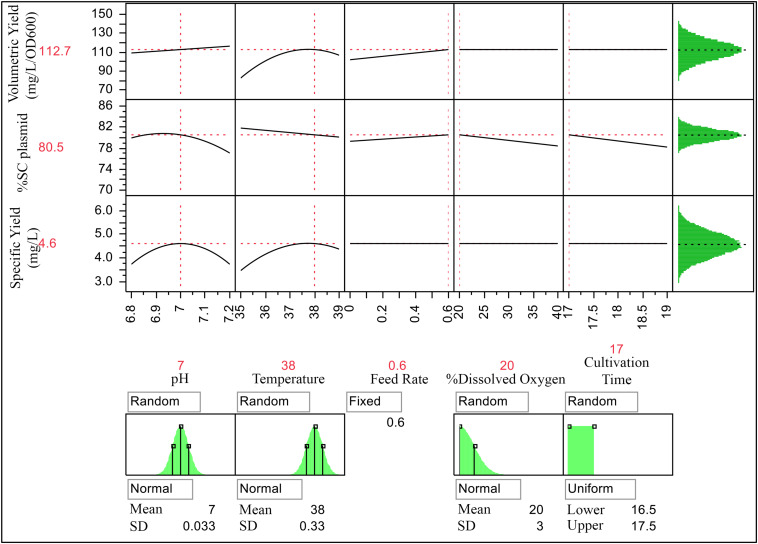
Prediction profiler for process optimization and simulation studies with Monte Carlo simulations of 100,000 runs with different data distribution types (shown underneath their respective response curves).

Results from these predictive modeling studies were then used to define ranges of CQA and PAs. Using JMP prediction profiler function, 100,000 runs were simulated with Monte Carlo simulation as its main advantage is that several types of probability distributions can be executed (Wang et al., 2007). Input PPs including temperature, pH, and %DO were modeled as a normal distribution, whereas feed rate and cultivation time were modeled as a fixed and uniform distribution, respectively. The simulations were performed with specified ranges of PPs listed in Table 6. Three key sources of variation, including the mathematical expression or model from the characterized process, variation of each factor at the targeted set point, and the residual variation not accounted for by the model, were included in this simulation. The residual variation is derived from the RMSE of the predictive model, the analytical method, and any other uncontrolled factor when building the predictive model. The population of CQA and PAs from these simulations is shown in Figure 4.

**TABLE 6:** Process parameters and distributions for process simulation.

**Process parameters**	**Set point and range**	**Distribution type**
Temperature (°C)	38 ± 1	Normal
pH	7 ± 0.1	Normal
Dissolved oxygen (%)	20 ± 10	Normal
Feed rate (mL/h/h)	0.6	Fixed
Cultivation time (h)	17 ± 0.5	Uniform

The simulations provided ranges for each attribute as follows 80.5 ± 1.12 of %SC plasmid DNA, 112.4 ± 10.39 mg pDNA/L of volumetric yield, and 4.6 ± 0.53 mg pDNA/L/OD600 of specific yield. From tolerance interval analysis with portion of population, the action range for PAs was set at 99.7% of the results, and the alert range was at 95.5% of the results, whereas the acceptable range for CQA was at 99.7% of the results. As a result, the action and alert limits of the model are provided and shown in Table 7. These values can then be applied for scale-up production of this vaccine.

**TABLE 7:** Ranges for CQA and PAs.

**Attribute**	**Acceptable range**	**Action range**	**Alert range**
**Critical quality attribute**			
%SC	77–84		
**Performance attribute**			
Volumetric yield (mg/L)		81–143	91–123
Specific yield (mg/L/OD600)		3.0–6.0	3.5–5.6

DSD can significantly reduce the development time and cost in the early stage of process development. The Monte Carlo simulations with predictive models are useful tools for process optimization, robustness study and subsequent process validation, and future commercial manufacturing. The utilization of knowledge gained from this study can be used to improve the process performance during the process development.

## Data Availability Statement

The raw data supporting the conclusions of this article will be made available by the authors, without undue reservation.

## Author Contributions

LH obtained grant support and conducted research and analysis. SN performed the experiments, statistic, and analysis. LH and SN wrote the manuscript. PK revised the manuscript. All authors contributed to the article and approved the submitted version.

## Conflict of Interest

The authors declare that the research was conducted in the absence of any commercial or financial relationships that could be construed as a potential conflict of interest.
